# Targeting lymphatic vessel functions through tyrosine kinases

**DOI:** 10.1186/2040-2384-2-13

**Published:** 2010-08-11

**Authors:** Steven P Williams, Tara Karnezis, Marc G Achen, Steven A Stacker

**Affiliations:** 1 Ludwig Institute for Cancer Research, Royal Melbourne Hospital, Parkville, Victoria 3050, Australia; 2 Department of Surgery, Royal Melbourne Hospital, University of Melbourne, Parkville, Victoria 3050, Australia; 3 Peter MacCallum Cancer Centre, St. Andrews Place, East Melbourne, Victoria 3002, Australia

## Abstract

The lymphatic vascular system is actively involved in tissue fluid homeostasis, immune surveillance and fatty acid transport. Pathological conditions can arise from injury to the lymphatics, or they can be recruited in the context of cancer to facilitate metastasis. Protein tyrosine kinases are central players in signal transduction networks and regulation of cell behavior. In the lymphatic endothelium, tyrosine kinases are involved in processes such as the maintenance of existing lymphatic vessels, growth and maturation of new vessels and modulation of their identity and function. As such, they are attractive targets for both existing inhibitors and the development of new inhibitors which affect lymphangiogenesis in pathological states such as cancer. RNAi screening provides an opportunity to identify the functional role of tyrosine kinases in the lymphatics. This review will discuss the role of tyrosine kinases in lymphatic biology and the potential use of inhibitors for anti-lymphangiogenic therapy.

## Introduction

A number of human diseases have been linked to abnormal or defective lymphatic vessels [1]. While the theory of anti-angiogenesis therapy has been extensively studied [2], the concept of targeting lymphangiogenesis to gain a therapeutic advantage in human disease is only a recent development [1]. Advances in our understanding of the molecular signaling pathways that control lymphatic vessel formation therefore provide an opportunity to explore the value of inhibiting these processes.

A good example of this is cancer biology, where the spread of tumor cells appears highly dependent on the vessels of the lymphatic system and the protein factors which drive their growth and differentiation [3]. As a consequence, therapeutic options which target these cellular pathways may provide a means to prevent growth or metastasis from the primary tumor. Therapeutics may be either anti-lymphatic (targeting functions of the existing vessels) and/or anti-lymphangiogenic (targeting the generation of new lymphatic vessels). An understanding of the key signaling components and cellular processes that are critical for lymphatic vessel function and growth is essential to enable the rational design of effective inhibitors.

One family of molecules, the protein tyrosine kinases, are known to be key drivers of angiogenesis [4], and studies have shown they also play a pivotal role in lymphatic biology/lymphangiogenesis [5]. In this review we explore the potential for this family of molecules to be used as targets for anti-lymphatic/anti-lymphangiogenesis and the ways in which we can gain insight into how these family members might contribute to key signaling pathways within the lymphatic endothelium.

### The lymphatic system in health and disease

While blood vessels carry oxygenated blood and nutrients to cells within the body, the lymphatic vessels act to maintain fluid homeostasis by draining excess fluid from the tissues, as well as contributing to immune surveillance and fatty acid transport. Fluid and cells released by the blood vessels are returned to the circulation via protein-rich lymph fluid that is drained by blind-ended capillaries in the superficial dermis. This is fed into the deeper, larger caliber lymphatic collecting vessels via lymph nodes and the thoracic duct and back to the circulation. All of these vessels have a specialized lining of endothelial cells. Both blood and lymphatic endothelial cells originate from common developmental precursors. Yet, it is now clear that the lymphatic endothelial cells differ in their molecular and physiological behavior to the "classical" blood endothelial cell [6,7].

Similarly, the endothelial cells of small lymphatic capillary vessels are distinct in function and gene expression from the lymphatic endothelial cells (LEC) that line the major collecting lymphatic vessels [8]. Interestingly, Baluk et al. recently described the presence of unique cell-cell junctions in lymphatic vessels [9]. They found lymphatic capillaries had discontinuous 'button-like' junctions that would allow flaps of the vessel to open and allow fluid entry. In contrast, collecting lymphatics had continuous 'zipper' junctions, yet in both vessel types the junctions appeared to have the same molecular components. How this organisation is achieved is unknown, but it presumably stems from the functional differences of the lymphatic vessel subtypes.

Florence Sabin's pioneering work of the early 20^th ^century mapped the development of the lymphatic vasculature by injecting blue dye into pig embryos, allowing the vessels to be visualized [10,11]. This foundation led to recent discoveries showing that early in embryonic development, lymphatic progenitor cells migrate away from the cardinal vein [12]. The process of developmental lymphangiogenesis proceeds with vessels sprouting from the lymph sacs formed from the progenitor cells. Many molecular signals are required to stimulate the correct lymphatic network development and maturation, some of which are discussed below.

**Figure 1:**
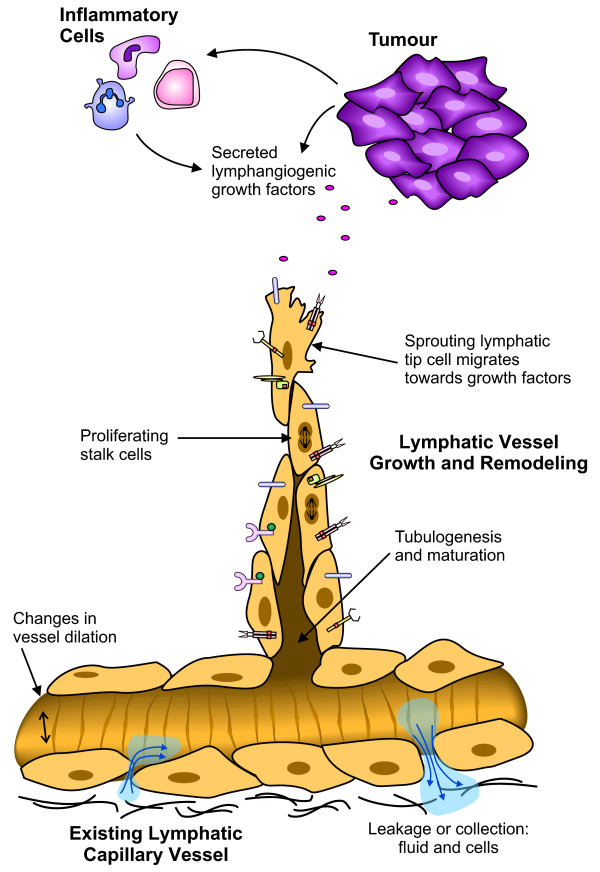
**Different functions of LECs in active lymphatic vessels**. This schematic outlines some of the cellular processes that occur in lymphatic vessels under pathological conditions such as cancer. In this diagram a tumor (and/or infiltrating immune cells) secretes factors that induce changes in the lymphatic vasculature. Growth factors binding to the different receptors expressed on the surface of the LECs may induce sprouting of new lymphatic vessels from existing lymphatic capillaries. The leading 'tip cell' detects a gradient of growth factors by means of cell surface receptors, and migrates towards the tumor. Behind the tip cell are the stalk cells, responding to proliferation stimuli. The formation of a lumen and maturation of the vessel is required to create a functional vessel. Other aspects of the vessel such as vessel dilation and vessel permeability to fluid and cells may also be altered. These characteristics may be exaggerated in the context of a tumor, to create the abnormal vessels often associated with cancer and enhance the ease with which lymphogenous metastasis occurs. Many of these responses are induced by signaling pathways involving tyrosine kinases.

In the context of human disease, both blood and lymphatic vessels play important roles. For example, in cancer, tumor progression relies on the angiogenic switch, or the induction of new blood vessel growth [13,14] for the supply of oxygen required for the tumor to grow. Blood vessels also provide a route for tumor dissemination to distant sites, via invasion of the bloodstream and homing to organs such as the brain, lungs, liver and bone [15]. Tumor angiogenesis (the growth of new blood vessels in a tumor) is therefore a valid target for cancer therapeutics. Recent work has shown that the lymphatic network also plays a central role in the metastasis of cancer, allowing spread to draining lymph nodes [16-18]. Clinically, many carcinomas are commonly seen to metastasize initially via the lymphatic vasculature to the lymph nodes [15], with the lymphatic vessels providing a key initial entry point for metastatic cancer cells. Numerous studies have shown a significant correlation between levels of the lymphangiogenic vascular endothelial growth factor C (VEGF-C), lymphatic vessel invasion, lymph node metastasis and/or overall survival (reviewed in [3,15,19]). Targeting the induction of tumor lymphangiogenesis (the generation of new lymphatic vessels within a tumor), and the signaling that drives functional changes in both new and existing lymphatic vessels (Figure 1), may help to prevent a route for tumor metastasis.

In addition to cancer, there are a range of pathological conditions associated with defective or abnormal lymphatic vessels. Lymphedema results from inadequate drainage of fluid from a limb, and can be primary or acquired. Primary lymphedema is rare, but patients are often found to harbour point mutations in key lymphatic genes such as vascular endothelial growth factor receptor 3 (VEGFR-3). Acquired lymphedema can be caused by damage or trauma to the lymphatic vessels (eg sentinel lymph node biopsy), or infection with the parasitic worms that cause filariasis (elephantiasis). Recent work by Tammela et al. [20] demonstrated that by stimulating the VEGFR-3 tyrosine kinase by treatment with the lymphangiogenic vascular endothelial growth factors C or D (VEGF-C or VEGF-D) it is possible to regenerate functional collecting lymphatic vessels in mice following lymph node dissection.

Lymphangioma or lymphangiectasia can result from a build up of fluid, causing an excessive dilation/distension of the lymphatic vessels that is not resolved. Patients (often children) may present with a group of skin lesions that discharge milky fluid, or cystic masses of the head, neck or genitals. Current treatments rely on compression bandages or surgery, although more recently sclerosing agents have been used with some success to induce fibrous obliteration of the vessel [21].

Therefore, understanding the biology of the lymphatics and lymphatic endothelium may provide new options for the treatment of diseases involving the lymphatics, such as cancer, lymphangioma, lymphedema and wound healing.

### Tyrosine kinases in vascular biology

#### Tyrosine kinase signaling in the cell

Protein phosphorylation is an important mechanism of reversible protein modification, allowing the cell to respond to a stimulus, and then turn the signaling pathway off when it is no longer required. The protein tyrosine kinase (PTK) family is a diverse group, that, when activated, catalyze the transfer of a phosphate group to specific tyrosine residues of their substrates. There are a total of 90 protein tyrosine kinases in humans, divided into the 58 receptor tyrosine kinases (RTKs) that bind extracellular ligands, and the 32 cytosolic (or non-receptor) tyrosine kinases [22,23]. The members of the PTK family are important signaling molecules in all eukaryotic cell types, enabling responses to growth, differentiation, cell-cell contact and apoptotic signals.

**Figure 2:**
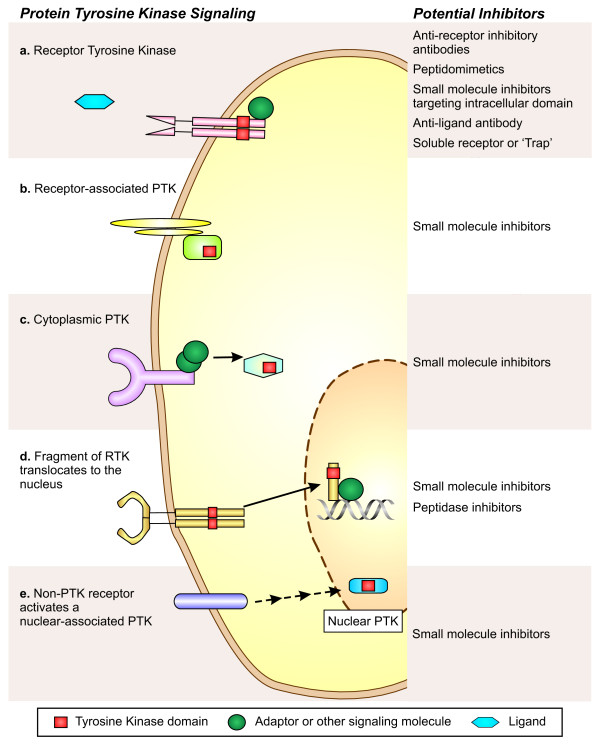
**Protein tyrosine kinase signaling pathways are potential targets in lymphatic endothelial cells**. Protein tyrosine kinases are a diverse group of proteins that act in different subcellular compartments of the cell. Outlined in the diagram are some examples of the types of signaling pathways involving tyrosine kinases: **a) **Receptor tyrosine kinases are expressed on the cell surface and bind their specific ligands. Ligand binding activates the intrinsic protein tyrosine kinase domain and triggers the signaling cascade. (For example VEGFR-3 signalling). **b) **Non-PTK cell surface receptors can be associated with cytoplasmic tyrosine kinases. The cytoplasmic tyrosine kinase may be brought into contact with the receptor by direct binding, an example of which is the JAK kinases. Alternatively, the PTK may be tethered to the plasma membrane allowing it to rapidly interact with the activated receptor. The Src family kinases act in this manner. **c) **Other cytosolic PTKs act downstream in the signaling pathway or more broadly throughout the cell. The c-Abl kinase is an example of a PTK with activity in various subcellular locations. **d) **In some circumstances, the intracellular domain of a receptor tyrosine kinase may be cleaved and translocate to the nucleus where it is able to phosphorylate different targets. (For example ErbB4 can signal in this manner). **e) **Nuclear associated tyrosine kinases are localized to the nucleus; their activity may be modulated in response to signaling pathways. (An example of this is the fyn-related kinase). The types of inhibitors that could be used to target each tyrosine kinase pathway are listed on the right of the figure.

PTKs allow the rapid transfer of a stimulus from the cell surface through to the nucleus; binding of a specific ligand by the cognate tyrosine kinase receptor causes dimerization and auto-phosphorylation at key residues of the intracellular domain. Once the receptor's kinase domain is activated this allows binding and phosphorylation of substrate proteins (Figure 2). Substrates are often activators or adaptors for cytosolic tyrosine kinases, such as the Src family kinases [24,25] which are directly involved in stimulus-induced signal transduction cascades. Cytosolic tyrosine kinases propagate the signal by driving the numerous downstream effector proteins, or inactivation of negative regulators. Non-receptor tyrosine kinases may be directly associated with a membrane receptor, located in the cytoplasm, or in the nucleus (Figure 2). It has also been reported that the intracellular portion of some RTKs can be cleaved, after which it can shuttle to the nucleus to alter gene expression [26].

Tyrosine kinases have been shown to play a role in the progression of cancer, where they resemble the prototypical oncogene. Aberrant expression, oncogenic fusion proteins or spontaneous mutations resulting in a constitutively active kinase domain are common in many PTK genes that have been linked to malignancy. This results in excessive signaling through a pathway in the absence of the ligand, which may drive the cell to proliferate unchecked and to ignore apoptotic stimuli. Given the important role of kinases as drivers of cancer, coupled with the reasonable ease with which they can be inhibited, it is not surprising that a significant proportion of anti-cancer therapeutics currently in trials are kinase inhibitors [27].

However, it is important to note that tumors also secrete ligands that act on RTKs expressed on the vasculature, thereby driving angiogenesis and/or lymphangiogenesis (Figure 1). As discussed below, preventing the stimulation of the (non-mutated) vascular tyrosine kinase pathways may also have significant therapeutic benefit [28].

#### The vascular endothelial growth factor receptors

In lymphatic endothelial cells (LECs) the vascular endothelial growth factor receptors (VEGFRs) are highly important tyrosine kinases due to their essential role in cell survival and proliferation (reviewed in [4,29]). VEGFR-1, VEGFR-2 and VEGFR-3 are mainly expressed on endothelial cells, with signaling through VEGFR-2 generally being angiogenic, and VEGFR-3 signaling being lymphangiogenic (Table 1). VEGFR-1 has been shown to regulate the migration of leukocytes, but its precise role in the vasculature is unclear. The receptors each have different affinities for the VEGF ligands; VEGF-A is a ligand for VEGFR-2 [30], as well as binding to VEGFR-1 [31], while VEGF-C and VEGF-D bind both VEGFR-2 and VEGFR-3 [4,32,33]. The other family members VEGF-B and placental growth factor (PlGF) are ligands for VEGFR-1 [4]. Binding of the VEGFs causes dimerization and autophosphorylation of the receptors, leading to activation of downstream kinases such as Ras-MAPK and PI3K-Akt pathways [34]. Both VEGF-C and VEGF-D overexpression has been shown to lead to lymphangiogenesis and lymphatic metastasis [17,18,35]. Significantly, deletion of both VEGF-C and VEGF-D together does not result in the embryonic blood vasculature defects seen in *VEGFR-3^-/-^*mice [36]. VEGF-C and VEGF-D are the only known ligands for VEGFR-3 [32,33], yet this finding suggests that there may be other additional ligands or a ligand-independent signaling mechanism. Work from Dixelius et al. [37] indicates that VEGFR-2 and VEGFR-3 can heterodimerize, and this may actually allow VEGF-A to induce signaling through VEGFR-3 [38]. However this VEGF-A induced VEGFR-3 activation is different to that seen with VEGF-C or VEGF-D; the two most carboxy-terminal tyrosine sites of VEGFR-3 are not phosphorylated by VEGFR-2 [37]. Interestingly, coreceptors such as Neuropilin-1 (NRP1) and NRP2 have been show to modulate the signaling pathways activated in response to VEGFs, enabling signals to elicit context dependent responses [39-41]. Both VEGF-C and VEGF-D have been shown to interact with NRP1 and NRP2 [42], but it is generally thought the neuropilins are unable to transduce VEGF signals without the VEGF receptors, although a recent study disputes this [43]. Clearly further work is required to elucidate the complexities of VEGFR-3 signaling.

**Table 1:** PTKs and their role in lymphatic biology

Gene	Role in lymphatic vessels	Inhibitors available*	Effect of pathway inhibition	References
*VEGFR-2*	Receptor for the VEGF family of ligands. Can also heterodimerize with VEGFR-3.	Yes	Secreted VEGFR-2 is a naturally occurring inhibitor of lymphatic vessel growth. However, Sorafenib^† ^did not block VEGF-C/D induced tumor lymphangiogenesis.	[132,133]
*VEGFR-3*	Predominant receptor for the lymphangiogenic growth factors VEGF-C and VEGF-D, transduces survival, proliferation and migration signals.	Yes	Cediranib^‡ ^blocks VEGFR-3 activity and inhibits lymphangiogenesis. Anti-VEGFR-3 antibody prevented tumor lymphangiogenesis with no effect on preexisting vessels.	[32,33,88,134-136]
*Tie1*	Not critical for lymphatic cell commitment during development, and no ligand has been shown.	None reported	*Tie1 *knockout mouse has lymphatic vascular abnormalities that precede the blood vessel phenotype.	[55]
*Tie2*	Receptor for Ang-1 and Ang-2, appears to control vessel maturation.	Yes	*Tie2^-/- ^*mice are embryonic lethal due to vascular defects. Inhibition of Ang-2 leads to tumor blood vessel normalization.	[49,50,137]
*EphB4*	Expressed on lymphatic capillary vessels, involved in vascular patterning, binds to the ephrinB2 ligand.	Yes	Mice expressing a mutant form of *ephrinB2 *lacking the PDZ binding domain show major lymphatic defects in capillary vessels and collecting vessel valve formation.	[60]
*SRC*	Signal transduction downstream from receptors.	Yes	Src inhibitor AZM475271 was effective at blocking VEGF-C driven lymphangiogenesis *in vivo*.	[103]
*FGFR3*	The ligands FGF-1 and FGF-2 promote proliferation, migration, and survival of cultured LECs. FGFR3 is direct transcriptional target of Prox1.	Yes	Knockdown of FGFR3 reduced LEC proliferation.	[47]
*IGF1R*	Both of the IGF1R ligands, IGF-1 and IGF-2, significantly stimulated proliferation and migration of primary lymphatic endothelial cells.	Yes	None reported.	[48]
*PDGFRβ*	The ligand PDGF-BB stimulated MAP kinase activity and cell motility of isolated lymphatic endothelial cells.	Yes	None reported.	[138]
*MET *	The ligand for c-Met, hepatocyte growth factor has lymphangiogenic effect, but it is unclear if c-Met is expressed on LECs.	Yes	May be indirect effect.	[45,46]

*For reviews detailing available inhibitors see [71,139]. ^†^Sorafenib inhibits B-Raf, PDGFRβ, VEGFR-2 and c-Kit. ^‡^Cediranib inhibits VEGFR-1, -2, -3, PDGFRβ and c-Kit.

#### Other tyrosine kinases in lymphatic biology

A number of other growth factor receptor families are suggested to play a role in LEC biology [44] and are summarized in Table 1. Hepatocyte growth factor, the ligand for the receptor tyrosine kinase c-Met, has been implicated in lymphangiogenesis, though whether this is acting directly on lymphatic endothelial cells or indirectly is not clear [45,46]. The lymphatic transcription factor Prox1 has been shown to upregulate fibroblast growth factor receptor 3 (FGFR3) [47]. This suggests that fibroblast growth factor (FGF) signaling is fundamentally important for LEC biology. The insulin-like growth factors 1 and 2 also induce LEC proliferation and migration [48] in a VEGFR-3 independent manner, presumably through insulin-like growth factor receptor 1 (IGF1R).

Another signaling system with specificity to endothelial cells consists of the Tie receptors (Tie1 and Tie2) and the Angiopoietin ligands (Ang1 and Ang2) [49,50]. This is probably also one of the more complex systems in terms of the contributions of each receptor. Both Ang1 and Ang2 bind the Tie2 receptor, but no ligand has yet been shown to bind Tie1. Knockout mice have shown that deletion of the Tie2 receptor results in lethality at embryonic day 10.5 (E10.5), due to vascular defects and cardiac failure [51,52]. A similar phenotype is seen in *Ang1 *knockout mice [53]. The *Tie1^-/- ^*mouse also dies during embryonic development [54], but at a much later stage (E13.5-E18.5), and displays altered lymph sac morphology [55]. These mice show ruptured microvessels and edema, indicating lymphatic dysfunction. This contrasts with the early lethality of *VEGFR-2^-/- ^*mice, which die around E9.0 [56]. Interestingly, the deletion of the Ang2 ligand does not cause death until postnatal day 14 [57], with significant defects in the lymphatic vasculature and retinal blood vessels. This clearly suggests an important role for Ang/Tie signaling in the latter stages of both blood and lymphatic vessel remodeling and the recruitment of mural cells to the mature vessels.

The Eph receptors are widely expressed RTKs, involved in patterning, morphology and intercellular adhesion. The Eph receptors are divided into A and B subtypes, and mostly bind only the membrane-bound ephrin ligands of the same type. EphB4 is selective for the ephrinB2 ligand, and in vascular patterning, it is this pair that are important [58,59]. EphB4 is found expressed on the venous endothelium, and ephrinB2 on the arteries. The lymphatic vessels also express these two molecules, with EphB4 distribution on the lymphatic capillaries, and ephrinB2 on the larger collecting lymphatics [60]. How these two molecules regulate vascular patterning is not entirely clear, however there is evidence to suggest VEGF regulates expression and the Notch pathway balances their effects [61]. Signaling through the Eph receptors is bidirectional; forward signaling is typical of receptor tyrosine kinases, following activation of the Eph receptor its kinase domain phosphorylates downstream targets such as Abl, though little is known about these pathways in endothelial biology. Reverse signaling occurs in the ephrin-expressing cell, and may involve Src family tyrosine kinases. The intracellular PDZ domain of ephrinB2 was deleted by Makinen et al. [60] and resulted in mice with lymphatic vasculature defects. Lymphatic capillary formation was affected, while collecting lymphatic vessels exhibited lack of valve formation. Additionally, alterations in the expression pattern of lymphatic vessel endothelial hyaluranon receptor 1 (LYVE-1) and abnormal smooth muscle cell recruitment indicated a failure to correctly specify the lymphatic vessel subtypes in the mutant mice. Potential PDZ binding partner proteins have been identified; it will be interesting to clarify the roles they play in vascular patterning.

Other tyrosine kinases are key regulators of molecules that control migration or survival. Cytosolic PTKs such as focal adhesion kinase (FAK), FES and FER are important for signals relating to interactions between the cytoskeleton and the extracellular matrix by integrins. Integrin α9 has been shown to be an important molecule in lymphatic endothelial cell biology, and is upregulated by the key lymphatic transcription factor Prox1 [62].

While most of the PTK family is now characterized to some extent, much of the endothelial cell work has been done in blood vasculature. In addition there are a number of PTKs that still are not well understood. It remains to be determined what, if any, role many of the PTKs have in LEC biology.

### Current strategies for targeting tyrosine kinases

Therapeutic targeting of PTKs has been approached from a number of angles, with varying success. Humanized monoclonal antibodies (mAb) raised against the extracellular domains of an RTK have been used. The first FDA approved PTK inhibitor was trastuzumab, a mAb directed to the HER2/neu RTK [63,64] for use against metastatic breast cancer. Since then, several others have made their way into the clinic; bevacizumab [65,66], and cetuximab [67,68] being the most significant examples. Monoclonal antibody inhibitors of RTKs act via prevention of receptor dimerization and ligand binding, and in some cases may cause receptor internalization and immune cell recruitment [64]. Antibodies generally allow much more specific blocking and thus have the advantage of specificity that small molecule inhibitors tend to lack. Inhibitory antibodies are however, only effective against cell surface receptors, and not against non-receptor tyrosine kinases.

Recent developments in medicinal chemistry and crystallography have led to the possibility of tailor-made small molecule inhibitors that are designed to fit perfectly into the active site of the kinase. These small molecules are able to enter the cell and it is therefore possible to target them to either the intracellular kinase domain of RTKs or the cytoplasmic tyrosine kinases. However one of the caveats of small molecule PTK inhibitors is that kinase domains are highly similar across the families, making selective inhibition difficult. This does mean that multiple pathways may be blocked simultaneously, which may have therapeutic benefit in some cases [27,69]. The disadvantage of a less selective small molecule PTK inhibitor is greater toxicity and risk of adverse effects. Some PTK inhibitors are well tolerated, however reported effects are anemia, rash, diarrhea, nausea, fatigue, weight loss and hypertension [70,71].

The prototype small molecule PTK inhibitor is imatinib; targeted to the chimeric protein that occurs in 95% of chronic myeloid leukemia patients as a result of the t(9;22)(q34;q11) translocation [72]. This fusion of the *BCR *gene to *ABL*, creates a constitutively active kinase [73]. Imatinib is able to selectively inhibit BCR-ABL driven cell proliferation at submicromolar concentrations, while having minimal effects on cells that do not have the translocation [74,75]. Imatinib's mechanism of action is now thought to be one of allosteric inhibition [76], binding to a site adjacent to the ATP pocket. More 'Type II' allosteric inhibitors are now being designed, that act by locking the kinase into an inactive state and preventing signal transduction (reviewed in [77]).

### Available strategies for anti-lymphangiogenesis therapy via PTK family

First proposed by Folkman in 1971 [2], anti-angiogenic therapy has now become accepted for cancer treatment [78]. Current strategies for targeting the blood vasculature are focused on inhibition of VEGF and/or blockade of the VEGFRs which activate the downstream pathways [71,79]. Bevacizumab, also known as Avastin (Genentech), is a monoclonal anti-VEGF antibody that has been approved in combination with chemotherapies for colorectal cancer, non-squamous non-small cell lung cancer, metastatic renal cell carcinoma and metastatic HER2-negative breast cancer [65,66]. Despite this, there is a risk of side effects such as gastrointestinal perforation, bleeding and impaired wound healing. Bevacizumab's exact mechanism of action is somewhat unclear, and while it may have some anti-angiogenic properties, the key may actually lie in the stabilization of tumor vessels. By normalizing the tumor vessels, the blood flow is increased and interstitial pressure is reduced allowing conventional chemotherapy better access to the tumor.

Other approaches have used soluble forms of VEGFR to create the 'VEGF-trap' (Regeneron), a recombinant chimeric decoy receptor which is in clinical trials [80,81]. Similarly, ImClone has developed inhibitory antibodies for VEGFR-1 [82] and VEGFR-2 [83-86], both of which are in clinical trials. A human neutralizing anti-VEGFR-3 antibody has also been generated [87]; in mouse experiments an equivalent antibody to mouse VEGFR-3 was shown to completely block tumor lymphangiogenesis with no effect on preexisting vessels [88] (Table 1). Soluble VEGFR-3 and antibodies targeted to VEGF-C and VEGF-D are in commercial development. Recently, several groups have had success creating peptidomimetics in a form that are resistant to degradation [89,90]. One of these is targeted to VEGFR-1 and NRP1, and appears effective at blocking angiogenesis in mouse models of retinopathy and cancer [90].

In contrast there are a large number of small molecule inhibitors available that inhibit VEGFR signaling [71]. However many of them also inhibit the activity of other related RTKs such as platelet derived growth factor receptors (PDGFRs), c-KIT and colony stimulating factor 1 receptor (CSF1R) due to similarity in the kinase site, and it is not uncommon to show activity against a wider range of kinases. The VEGF receptor inhibitors that have been FDA approved as chemotherapeutics are sorafenib (Bayer) [91,92], sunitinib (Pfizer) [93-95] and pazopanib (GlaxoSmithKline) [96]. One of the commonly seen issues associated with all anti-VEGF treatments is resistance, as alternative proangiogenic pathways are switched on. Small molecule inhibitors that target multiple pathways (e.g. VEGFRs, FGFRs and PDGFRs) simultaneously may avoid this problem, but also increase the risk of associated side-effects. Sorafenib was originally designed to inhibit B-Raf, and was found to be effective in renal and hepatocellular cancers. However, this is now attributed not to the inhibition of B-Raf, but to its activity against VEGFR-2 and PDGFRβ [69]. This leads to blockade of angiogenesis through VEGFR-2, and PDGFRβ inhibition prevents the recruitment of pericytes for vessel stabilization. Recently Murphy et al. [97] reported a second generation 'Type II' inhibitor, designed to be highly selective for PDGFRβ and B-Raf. Oral administration of this compound was able to suppress growth of orthotopic kidney and pancreatic tumors in mice, with significant anti-angiogenic effects.

Eph-Ephrin signaling is a promising anti-angiogenic/anti-lymphangiogenic target. A number of small molecule inhibitors are available [59], including EXEL-7647. EXEL-7647 is currently in clinical trials, and inhibits epidermal growth factor receptor (EGFR), ErbB2, VEGFRs and EphB4 [98,99]. Other inhibitors in the form of peptidomimetics, inhibitory monoclonal antibodies, and soluble receptors are being tested [59]. It also remains to investigate in more detail the contribution of other Eph receptors to vascular biology; EphA2 signaling has been shown to contribute to tumor angiogenesis, while the ligand ephrinA1 can be upregulated by VEGF [100]. This complex field of Eph signaling, if well understood, could give rise to a range of useful therapeutics.

The nine Src family kinases are cytoplasmic PTKs closely associated with the cell membrane and both RTKs and non-PTK receptors (Figure 2). Src family kinases mediate signal transduction pathways relating to many critical functions of a cell; proliferation, apoptosis, cell adhesion and migration [25]. A number of small molecule inhibitors are available, and several are in clinical trials [25]. Inhibitors of Src family kinases may be useful both to reduce the expression of growth factors in tumor cells [101], as well as having direct effects on the endothelium. Src is known to interact with VEGF receptors, and a selective Src inhibitor significantly reduced human umbilical vein endothelial cell (HUVEC) proliferation and migration *in vitro *[102]. Recently Ischenko et al. showed that the Src inhibitor AZM475271 was effective at blocking VEGF-C driven lymphangiogenesis *in vivo *[103] (Table 1). Previously this inhibitor had been demonstrated to have potent anti-tumor and anti-angiogenic effects in mouse pancreatic cancer models [104]. This suggests a common mechanism that could be targeted to simultaneously block lymphangiogenesis, angiogenesis and tumorigenesis.

Currently there are no PTK inhibitors specifically targeting the lymphatics. Even VEGFR-3, which was thought to be specific to LECs, has now been shown to be expressed at the leading edge of sprouting blood vasculature [105]. Therefore this remains an attractive target for dual inhibition of blood and lymphatic growth [105]. Encouragingly, it was recently shown that inhibition of the coreceptor NRP2 specifically blocked lymphatic vessel sprouting and migration but did not affect cell proliferation [40,106]. As many of the trials of PTK inhibitors have been focused on anti-angiogenic efficacy, it remains to be determined whether any possess significant anti-lymphangiogenic potential. Evaluation of specific inhibitors will be required to identify those that have activity in *in vitro *and *in vivo *lymphangiogenesis assays.

### Identifying new targets for anti-lymphatic treatment

In order to identify new targets for anti-lymphangiogenic treatments efficiently, screening strategies must be successfully employed. The recent and exciting advent of RNAi technology and high throughput screening systems have allowed researchers to investigate the functional importance of a large number of genes in *in vitro *assays [107-109]. RNAi screens have been successfully used to identify new molecules involved in many processes including cell cycle [110,111], apoptosis [112], endocytosis [113], cell migration [114-116], morphology [117], neural outgrowth [118] and drug resistance [119]. It has also been useful in dissecting molecular pathways to identify new regulators and downstream mediators [120-124]. Yet this powerful technique has hardly been utilized in studying endothelial cell biology. RNAi screens could potentially identify new anti-lymphangiogenic targets by screening for LEC migration and proliferation genes, or by screening for regulators of key molecular pathways. Many commercial companies now offer siRNA libraries covering the human kinome, making RNAi screening feasible for research laboratories. RNAi screens are primarily considered a target identification tool, as there are still some obstacles to be overcome to the clinical application of siRNA therapy. In addition, hits from a screen may not be easily druggable, or a drug may give a different phenotype to the siRNA [27]. Nonetheless, a recent study does show that there are feasible and effective methods for specific targeting and delivery of siRNAs in humans [125], suggesting the RNAi screen may soon be used as a direct therapeutic agent identification tool.

High throughput screening of chemical libraries offers the opportunity to screen thousands of compounds to identify small molecule inhibitors of a cell process of interest [126-129]. If a key kinase target is known, the assay readout can be set to indicate whether the compound is on-target [130]. Chemical library screens are commonly performed *in vitro*, however the use of model organisms such as Xenopus and Zebrafish has enabled high throughput chemical screens to be carried out *in vivo*. Kälin et al. recently screened 1280 compounds looking for modulators of angiogenesis and lymphangiogenesis in Xenopus [131]. Interestingly, several compounds known to inhibit tyrosine kinases were identified as having selective anti-lymphangiogenic activity.

Alternatively, once a target has been identified, rational drug design can be used to develop a compound that binds with high specificity [77]. This approach has been used to create drugs such as imatinib, but also more recently a selective inhibitor of both B-Raf and PDGFRβ [97]. Finding the balance between highly selective compounds and still inhibiting the multiple necessary pathways to see maximal effect without causing severe side-effects will require a combination of approaches. RNAi screening allows the entire genome to be screened, including the thousands of virtually unannotated genes. Similarly, chemical libraries now comprise hundreds of thousands of compounds, many of which are unknown. These platform technologies may soon provide targets and lead compounds, and eventually give rise to reagents targeting protein tyrosine kinases for anti-lymphangiogenic therapy that have clinical application.

## Conclusions

The recent renaissance in lymphatic endothelial biology has led to a better understanding of the important role these vessels play in health and disease. It is now apparent that specific targeting of protein tyrosine kinases is an effective way to elicit anti-angiogenic responses in the context of cancer therapy. Similar approaches could be used to target lymphatics to prevent metastasis, while in other pathological conditions such as lymphedema, targeted therapy may be used to restore their growth and subsequent function. Some of these treatments have been developed to existing targets such as the VEGFRs and their ligands. Further testing will be required to fully determine their efficacy, but there are also potentially many novel targets not yet discovered or not currently associated with lymphatic biology.

## Competing interests

SAS and MGA are consultants for Vegenics Ltd, a company which develops inhibitors of VEGF receptors.

## Authors' contributions

SPW, TK, MGA and SAS were involved in preparation of the manuscript. All the authors read and approved the final manuscript.
